# A deliberative dialogue as a knowledge translation strategy on road traffic injuries in Burkina Faso: a mixed-method evaluation

**DOI:** 10.1186/s12961-018-0388-8

**Published:** 2018-11-20

**Authors:** Esther Mc Sween-Cadieux, Christian Dagenais, Valéry Ridde

**Affiliations:** 1 Department of Psychology, University of Montreal, P.O. Box 6128, Centre-Ville Station, Montreal, QC H3C 3J7 Canada; 2 IRD (French Institute for Research on sustainable Development), CEPED (IRD-Université Paris Descartes), Universités Paris Sorbonne Cités, ERL INSERM SAGESUD, Paris, France; 3 University of Montreal Public Health Research Institute (IRSPUM), 7101 Avenue du Parc, 3rd Floor, Montreal, QC H3N 1X9 Canada

**Keywords:** Deliberative workshop, Knowledge translation, Research dissemination, Research utilisation, Research use, Evaluation, Road safety, Public health, Burkina Faso, West Africa

## Abstract

**Introduction:**

Deliberative dialogues are increasingly being used, particularly on the African continent. They are a promising interactive knowledge translation strategy that brings together and leverages the knowledge of diverse stakeholders important to the resolution of a societal issue. Following a research project carried out in Burkina Faso on road traffic injuries, a 1-day workshop in the form of a deliberative dialogue was organised in November 2015. The workshop brought together actors involved in road safety, such as researchers, police and fire brigades, health professionals, non-governmental and civil society organisations, and representatives of government structures. The objective was to present the research results, propose recommendations to improve the situation and develop a collective action plan.

**Method:**

To better understand the workshop’s utility and effects, a mixed-method evaluation was conducted. Data were obtained from two questionnaires distributed at the end of the workshop (*n* = 37) and 14 qualitative interviews with participants 6–10 weeks after the workshop. Descriptive statistics were used to analyse the quantitative data, and a thematic analysis was conducted for the qualitative data.

**Results:**

The data revealed several positive impacts of the workshop, such as the acquisition of new knowledge about road safety, the opportunity for participants to learn from each other, the creation of post-workshop collaborations, and individual behaviour changes. However, several challenges were encountered that constrained the potential effects of the workshop, including the limited presence of political actors, the lack of engagement among participants to develop an action plan, and the difficulty in setting up a monitoring committee following the workshop.

**Conclusion:**

While the deliberative workshop is not the standard format for reporting research results in Burkina Faso, this model should be reproduced in different contexts. This interactive knowledge translation strategy is useful to benefit from the experiential knowledge of the various actors and to encourage their involvement in formulating recommendations.

## Background

The process of moving from research to action is complex and multifactorial. However, the use of research is important for improving health policy, interventions and decision-making, especially in low-income countries, where health indicators continue to be a concern and equity is sometimes neglected. Knowledge translation (KT) aims to produce, synthesise, disseminate and share research-based evidence (RBE) to support its application [1, 2]. KT can provide mechanisms by which researchers and users can interact to combine scientific knowledge with experiential knowledge [3]. With this approach, users can be included in developing, producing, interpreting and applying RBE [4]. Integrating the knowledge of all stakeholders involved in resolving a health issue is essential to producing concrete and realistic recommendations that are adapted to the local context and based on research results [5, 6]. However, thus far, few studies have evaluated interactive KT strategies in West Africa [7–9].

Deliberative or policy dialogues are increasingly being used, particularly on the African continent. These dialogues “*allow research evidence to be considered together with the views, experiences and tacit knowledge of those who will be involved in, or affected by, future decisions about a high-priority issue*” [10]. They are used to draw on the knowledge of various key stakeholders involved in addressing a societal issue, such as researchers, policy-makers, practitioners and civil society organisations (CSOs) [11–14]. The value of this deliberative process lies in the fact that RBE is just one of the factors influencing decision-making, and that a variety of actors, not just policy-makers, can also act and contribute significantly to decision-making processes [10]. According to best practices [15], a deliberative dialogue should address a priority policy issue, look at it from different angles and examine its impact on different groups, foster discussions around various solutions to resolve the problem, and consider the feasibility of proposed solutions. To ensure the dialogue is based on the most relevant knowledge, one or more research briefs should be prepared to consolidate the main research findings and present possible solutions [16]. These briefs should be distributed at least 2 weeks before the event [10]. While a deliberative dialogue is not expected to reach a consensus on any decision [17], it could conclude with the stakeholders making commitments to undertake a set of concrete actions.

### The case of road safety in Burkina Faso

The African continent has the highest road accident mortality rates in the world [18]. Moreover, according to the WHO report on road safety in the African region [19], official data tend to be vastly underreported and discrepancies are often observed between data sources [20]. Among young people aged 15–29 years, road accidents are the leading cause of death. According to WHO, the efforts being made to meet the target set by the sustainable development goals, which is to halve the number of road traffic deaths and injuries, are insufficient [18]. In Burkina Faso, a landlocked country in West Africa, road accidents represent a significant public health burden. According to the National Police and Gendarmerie, 1125 fatalities at the crash scene were reported in 2013 [19]. In the same report, WHO data estimated the mortality rate at 30 per 100,000 population [19]. In Ouagadougou, the country’s capital, a steady increase in the number of accidents has been observed since 2005 [21]. However, very few studies have been performed on the subject in this region of the world, and the data available on road accidents are not always rigorous [22].

In 2008, the government of Burkina Faso set up the National Road Safety Council and the National Road Safety Office (ONASER), with the mission to ensure the smooth flow of road traffic, improve the road network and promote road safety. More than 20 associations working in the field of safety promotion and road safety education joined forces in 2012 (FAPSER-BF) for better coordination of community actions. Despite the creation of these structures, the development of a 2011–2020 action plan, and the implementation of several actions to improve the situation, road accidents remain a public health issue, requiring better coordination of forces and adequate funding. In addition, road safety law enforcement is weak in the country and the helmet wearing rate remains low among two-wheeled vehicle users [18, 19].

In 2015, a research project was conducted on road traffic injuries in Ouagadougou as the result of a collaboration between the Institut de recherche pour le développement of France, the Burkina Faso National Police, and the Centre Hospitalier Universitaire Yalgado Ouédraogo, as part of a research programme funded by the Canadian Institutes of Health Research. The purpose of the study was to test the effectiveness, acceptability and capacity of a surveillance system to assess the number of accidents and their consequences on the health of accident victims [21]. This study counted the accidents reported to the police for 6 months, identified the most accident-prone areas, estimated mortality, morbidity and disabilities resulting from road accidents, identified vulnerable road users and, finally, monitored the treatment of the injured. The collaborative approach of the study and the innovative monitoring system set up to collect accident data were strengths of the project [20]. In less than 7 months, police officers recorded 2752 crash scenes, involving 1338 injuries and 25 deaths [23]. During the same period, the results from the survey conducted by the hospital emergency trauma services showed that 1867 victims were admitted to the main hospital and 47 deaths were reported [20, 24]. More than 80% of the injured were users of two-wheeled vehicles. Furthermore, the study also showed that 26% of the injured still experienced disabilities 30 days after the accident [20].

### A deliberative dialogue workshop on road safety

A dissemination workshop, in the form of a deliberative dialogue, was organised in November 2015. The main objectives were to report the study’s results and, based on these findings, to propose recommendations to address the situation. To improve on the traditional format of research dissemination workshops [25], the researchers sought to create a more participatory and egalitarian dialogue by building on the diversity of knowledge, inviting a wide variety of stakeholders involved in road safety, limiting the number of scientific presentations to allow more time for group work, and encouraging the development of an action plan to implement the recommendations resulting from the RBE and deliberations. Several structures were represented, including the police, fire brigades, non-governmental organisations (NGOs) and CSOs involved in road safety, as well as research centres, the ministries of Scientific Research, Innovation, and National Education and Literacy, and ONASER. Nearly 60 people attended the opening and 45 people participated from start to finish. Additionally, three research briefs were prepared by the researchers and distributed to participants 1 week prior to the workshop. These were reviewed by KT specialists to ensure the content was clear, accessible and applicable [23, 24, 26, 27].

The workshop was conducted in three phases (Box 1). First, the researchers presented the main results of the study, followed by a question period. Presentations were based on the content of the briefs but provided more detail. Subsequently, the 11 recommendations arising from the study findings and presented in the briefs were discussed. The participants voted by a show of hands on each recommendation’s relevance and feasibility in Burkina Faso. Voting cards in the colours of traffic lights were distributed. The green card meant the recommendation was feasible, the yellow card meant it was considered moderately feasible, and the red card meant it would be difficult to implement. In the second phase, participants were divided into three subgroups (health and health transport, law enforcement, civil society). A framework was designed to structure the discussions. Each subgroup was asked to identify recommendations upon which they could act, to adapt them or formulate new ones, and to propose actions for context-appropriate implementation. Third, in the plenary session, each group presented its recommendations and reflections on their implementation, and then participants discussed their post-workshop commitments. The workshop was facilitated by a neutral and qualified KT specialist familiar with the issue, who had not been involved in the research project.

To know the relevance, acceptability and impact of this deliberative workshop in the Burkinabè context, an evaluation was conducted. This article presents the results of the evaluation, whose objectives were to (1) assess participants’ reactions to the conduct and content of the deliberative workshop and the research briefs distributed; (2) measure their intention to use the knowledge presented at the workshop; (3) measure their use of this knowledge in the months following the workshop; and (4) make suggestions for improving the follow-up and implementation of recommendations coming out of the workshop.

## Methods

### Conceptual framework

**Fig. 1:**
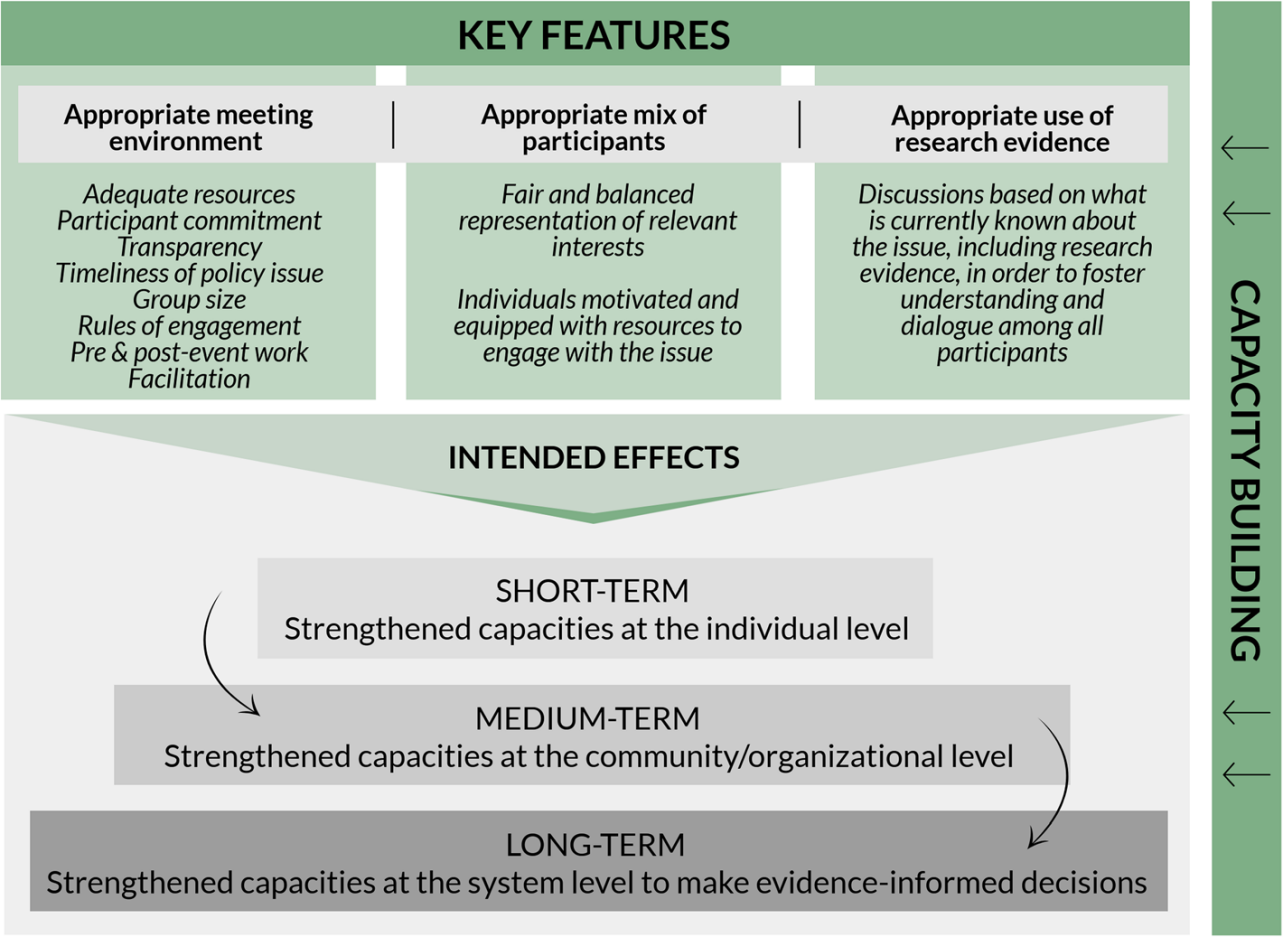
Conceptual model of deliberative dialogue as a KT strategy adapted from Boyko et al. [17]

The conceptual model developed by Boyko et al. [17] guided the organisation and evaluation of the deliberative workshop. This model identifies three key elements for organising a deliberative workshop, namely a supportive environment, the presence of a variety of participants and the use of research results in deliberations (Fig. 1). The expected short-term effects of a deliberative workshop are, first, an improvement in knowledge and skills at the individual level (e.g. what is known about the issue and how it might be addressed) and then an improvement at the organisational level to better respond to the issue collectively [17]. Long-term objectives are to promote access to research-based knowledge, to support stakeholders and, ultimately, to develop a culture that values evidence-based decision-making [17].

### Data collection

A mixed-method evaluation design was favoured [28] since the use of different types of complementary data increases the validity and credibility of the results [29]. A convergent design was selected, wherein qualitative and quantitative data were collected and analysed independently, but the findings were compared and combined in the interpretation [30]. The quantitative and qualitative results were compared to identify key similarities or discrepancies. The qualitative component was nevertheless predominant, since the interview data were used to explain, clarify and refine several quantitative results. The qualitative component also collected additional data (e.g. new knowledge gained, ways to improve the workshop, etc.) that would be complex to quantify.

#### Quantitative component

The quantitative component assessed participants’ reactions after the workshop (objective 1) and measured their intention to use the knowledge imparted (objective 2). The instruments used to meet these objectives were two questionnaires, which assessed (1) the overall evaluation of the deliberative workshop (12 questions) and (2) the intention to use the knowledge (14 questions). Participants rated the various questions on a 7-point Likert scale ranging from ‘strongly disagree’ (1 point) to ‘strongly agree’ (7 points). The questionnaires were completed at the end of the day (*n* = 37/45; 82%). The data were analysed using descriptive statistics.

##### Questionnaire on the overall evaluation of the workshop

The questions regarding participants’ overall evaluation of the workshop focused on their achievement of personal objectives, the quality of the discussions and of the workshop organisation, and the usefulness of the knowledge transmitted. Three additional qualitative questions concerned the elements most and least appreciated by participants, as well as suggestions for improving the KT process.

##### Questionnaire on intention to use the knowledge

The questions on the intention to use knowledge come from a tool based on the theory of planned behaviour [31]. This tool was developed and partially validated by Boyko et al. [32] with policy-makers in Canada. This theory postulates that the intention to use research is a predictor of actual use. The questionnaire measures four constructs, namely intention to use, attitudes, subjective norms (social pressure), and perceived control over behaviour. The tool has good internal consistency (Cronbach’s alpha ranges from 0.68 to 0.89) [32] but has not been validated in the Burkinabè context.

#### Qualitative component

The qualitative component was used to clarify and deepen participants’ assessments following the workshop (objective 1), to identify the knowledge use reported by participants (objective 3), and to understand how to improve the follow-up and implementation of recommendations (objective 4).

Semi-structured interviews were conducted 6–10 weeks after the activity with 14 participants. Three respondents had not attended the workshop to the end, but it was still important to obtain their views on KT because of their professional status. In-depth contrast sampling was used to ensure that at least one representative from each relevant group was present in the sample for the purpose of the survey [33]. This diversity of actors made it possible to highlight the different points of view. The respondents consisted of representatives of international NGOs (*n* = 2), a health professional (*n* = 1), a police chief (*n* = 1), a driving school manager (*n* = 1), youth CSO leaders (*n* = 2), road safety CSO leaders (*n* = 2), representatives of a government structure/ministry (*n* = 2), representatives of a national research centre (*n* = 2), and a road infrastructure professional (*n* = 1). The interviews lasted 45 min on average and were conducted by the first author (EMC), since both co-authors (CD, VR) were involved in the organisation of the workshop.

The interview grid was based mainly on the elements identified in the conceptual model of Boyko et al. [17] (Fig. 1). Several themes were addressed, such as the respondents’ evaluation of the researchers’ presentations and the discussions, their opinion on the group composition and dynamics, the usefulness of the workshop and its potential impact, any obstacles to implementing the proposed recommendations, the monitoring committee, ideas for improving the workshop, etc. The qualitative data were analysed using the thematic analysis approach defined by Paillé and Mucchielli [34]. The interviews were first coded using the themes identified in the interview grid. The different themes that emerged during the meetings were also coded. In addition, the different types of research use were coded, as follows: ‘conceptual use’ (change in understanding, attitudes or conception of an issue), ‘instrumental use’ (change in behaviour, practice or decision-making), ‘persuasive use’ (influencing decisions, legitimising positions or actions, convincing others to take a position), and ‘process-related use’ (changes due to involvement in the research or evaluation process) [35, 36]. Once the main themes were coded, the perceptions of the various respondents were compared to obtain a complete and nuanced portrait.

## Results

The results are presented in line with the evaluation objectives, namely (1) evaluation of the workshop and research briefs; (2) intention to use the knowledge; (3) types of use reported; and (4) follow-up and implementation of workshop recommendations.

### Evaluation of the workshop and research briefs

All respondents acknowledged the relevance of a workshop not only to report the results of the research, but also to compare the researchers’ proposals with the realities on the ground. In general, the workshop was appreciated and met the participants’ expectations (Table 1), which were to acquire knowledge on road accidents, to find out the results of the study and to establish relationships with other actors in the field.

**Table 1:** Mean scores for items in the questionnaire on reactions (*n* = 37)

Items evaluated	Min.	Max.	Mean	Standard deviation
I am satisfied with the quality of the presentations	5	7	6.47	0.65
The content presented was understandable	5	7	6.39	0.69
The content of this workshop met my expectations	5	7	6.30	0.62
The information presented will be useful to me in my work	4	7	6.11	1.02
I am satisfied with the subject matter covered	1	7	5.97	1.36
I am satisfied with the quality of the debates	3	7	5.86	1.00
The ideas presented at the workshop were new to me	1	7	3.74	2.28

#### Research briefs

Participants rated all three research briefs positively and recognised the value of having access to these documents. Several stressed the importance of visual presentation in communicating statistical data (graphs, location maps, tables). Very few items were proposed to be modified in the research briefs. However, as the briefs were relatively short, several participants suggested making the content more explicit (e.g. providing social profiles of accident victims, presenting more detail on the causes and circumstances of accidents, developing a brief on socio-anthropological analysis). Suggestions for improvement were limited to involving the various road safety stakeholders in preparing the briefs and adding a target audience to the recommendations. Many stressed the importance of sending briefs out a few weeks before the workshop.

#### Group climate and dynamics

The workshop brought together a wide variety of actors involved in road safety (prevention, accident investigation, care of the injured, healthcare, rehabilitation). This diversity was appreciated, as this was the first comprehensive consultation effort. The participatory atmosphere, and the discussions and debates among the participants, were the elements most appreciated by respondents to the evaluation questionnaire. Participants also said they benefited from networking to get to know the structures active in the field. A few reported having created collaborations there that were consolidated following the workshop.

Several respondents said they understood the realities on the ground better as a result of the discussions following the researchers’ presentations and during the group work. The participants’ diverse experiences led them to compare ideas that could bring about changes in practices. While the workshop generated some more lively discussions between certain structures, a climate of respect and listening prevailed.

“*People didn’t come to the workshop to defend their approach, but rather to ask questions about how the others were thinking. It was really interactive.*” (NGO representative)

However, several respondents noted the absence of decision-makers or ministry representatives. As the workshop was held 1 week before the presidential elections, some said the timing was not ideal. Several stressed the importance of making additional efforts to ensure their presence such as by sending out invitations well in advance, given the time frames involved in ministry procedures, going to them to extend the invitation or presenting them with the key findings of the study in advance. The impact of the workshop can be limited if decision-makers are not present for the summary of recommendations.

“*We communicate to our hierarchy, but it doesn’t have the same impact as it does when the decision-maker is there and sees the presentation for himself. It’s not the same when it comes through another person.*” (Health professional)

#### Presentations of research results

All respondents felt the researchers’ presentations were accessible. The qualitative data from the evaluation questionnaire also showed that the second most appreciated aspect was the clarity of the information provided during the presentations. However, it should be noted that several participants were involved in the research process and that they already knew the main results presented. As Table 1 shows, the average score for new knowledge transmitted was the lowest (3.74/7). Participants were particularly interested in the examples from neighbouring countries and in the images used to create road safety awareness in the presentations. The fast pace of the presentations was acceptable, but some respondents noted that too little time was allocated for discussion following each large group presentation. To balance the contribution of each group of actors, some suggested that speaking time should also be given to certain structures during the morning to present their road safety activities and raise the key issues.

Some respondents suggested that the objectives of the deliberative workshop should be clearly explained at the start of the day, including the objective of developing a collective action plan to implement the recommendations emerging from the workshop. Many had expected it to be a traditional research dissemination workshop in which participants generally leave without having any task to perform or follow-up to do.

#### Working in subgroups

The majority of respondents appreciated working in subgroups during the afternoon to reflect concretely on the actions to take and to share their respective concerns. Having this opportunity for direct conversations allowed several participants to change their views on certain partners.

“*We used to wonder why the police didn’t penalise people more, but now, listening to them give their perspectives, we were given the reasons for this behaviour. It is rare that we have the opportunity to exchange in this way.*” (Driving school instructor)

However, several civil society actors proposed dividing participants into diverse groups to take greater advantage of the variety of actors present. It was also proposed to organise pre-workshop consultations with each group of stakeholders so that the discussions could be more in-depth and applied on the workshop day. The grid used to structure the group work was useful, mainly because it helped refocus the discussions around the feasibility of the recommendations.

“*Sometimes people talk about possible solutions but with no means to put them in place, but in this case, some things were eliminated because they couldn’t be accomplished... The grid focused on how to achieve the objective, and that was useful.*” (NGO representative)

#### Synthesis and action plan

The collective synthesis following the group work did not generate much discussion and deliberation. This result was consistent with the evaluation of the debates in the quantitative questionnaire, as this item obtained lower scores (Table 1). Additionally, the synthesis of the recommendations did not elicit the desired commitment to develop an action plan and set up a monitoring committee. A ministry representative spoke about the need to consult upper line managers before engaging in such initiatives and to respect the procedures of their parent organisation. Thus, this request for mobilisation to implement the recommendations appeared to have surprised the participants:

“*The people present were shaken because the workshop had been going very well. There were reflections, which happens in one workshop out of four, in my opinion... But the request for mobilisation* [that emerged] *without notice, although quite logical and well presented, was not necessarily compatible with the logics, the rhythms of Burkina Faso, and with the absence of any existing rudimentary platform in road safety.*” (NGO representative)

Table 2 summarises the evaluation results in relation to the key features to be respected in deliberative workshops, as proposed by the conceptual model of Boyko et al. [17, 37] (Fig. 1).

**Table 2:** Summary of the characteristics of the deliberative workshop

Key features		
Appropriate meeting environment	Appropriate mix of participants	Appropriate use of research evidence
- Appropriate group size - Adequate facilitation - Transparency respected (objectives and funding) - Participants not well prepared for the deliberations (clarify expectations and procedures beforehand) - Lack of leadership and limited resources for following up on the workshop (research project) - Timing: issue not yet on the political agenda at the time of the workshop	- Participants representative of the diversity of sectors involved in road safety - Low attendance by policy-makers at the afternoon deliberations - Participants motivated, but limited in their power to act (transmission to the hierarchy)	- Three research briefs that summarised the main results of the study - Presentations that were clear, concise and tailored to the audience - Presentation on the current status of the situation (e.g. legislation, comparisons with other countries) - Recommendations proposed by the researchers and discussed collectively to structure and feed the discussions

### Intention to use the knowledge

Table 3 presents the scores obtained on the different items of the questionnaire measuring the intention to use knowledge. Generally, respondents intended to use the knowledge (6.03/7) and already envisioned an opportunity to do so (6/7). The items with the highest scores were those related to participants’ attitudes (mean 6.67/7), which may support the acceptability of the study results. However, items measuring respondents’ perception of their control over knowledge use scored lower (3.63/7 to 4/7), but with significant variation in scores (SD 2.17). In analysing the sociodemographic characteristics, it can be seen that many respondents with higher scores (perception of less control) were trainees, students, safety officers or driving school trainers. This is consistent with the importance of reporting to their line managers, as mentioned above. This result is also consistent with the qualitative data, as many respondents deplored the absence of senior decision-makers, ministry representatives and government authorities at the deliberations.

**Table 3:** Respondents’ level of agreement with the intention to use knowledge (*n* = 37)

Constructs	Items	Min.	Max.	Mean	Standard deviation
Behavioural intentions	I intend to use it	4	7	6.03	0.91
	I expect to use it	4	7	6.00	0.93
Subjective norms	It is expected of me that I use it (agree/disagree)	1	7	5.53	1.54
	I feel under social pressure to use it (agree/disagree)	1	7	4.59	1.54
	People who are important to me want me to use it (agree/disagree)	1	7	5.06	1.63
	People who are important to me think that I should/should not use it	4	7	5.89	1.02
Perceived behavioural control	I am confident I could use it (agree/disagree)	3	7	5.86	1.06
	It is easy/difficult for me to use it	2	7	5.82	1.22
	The decision to use it is beyond my control (agree/disagree)	1	7	3.63	2.17
	Whether or not I used it is entirely up to me (agree/disagree)	1	7	4.00	2.17
Attitudes	Using it is beneficial/harmful	4	7	6.69	0.78
	Using it is good/bad	4	7	6.66	0.64
	Using it is pleasant/unpleasant	4	7	6.59	0.70
	Using it is helpful/unhelpful	5	7	6.74	0.51

### Types of knowledge use

The effects reported by participants after the workshop are described in Table 4 and more specific examples of different types of knowledge use are presented in the following sections. The main perceived impact of the workshop is that participants got access to evidence and gained a better understanding of the road accidents situation. Several participants also shared the main results in their network and some were motivated to organise public prevention activities, especially with young people. Moreover, many impacts reported by respondents stem more from their participation in the deliberative process and the networking. For example, the workshop allowed stakeholders to become familiar with local actors, to create partnerships, and to develop interest and motivation in KT through the organisation of subsequent workshops.

**Table 4:** Main effects reported by participants after the workshop

Type of knowledge use	Examples of knowledge use reported by participants
Conceptual	- Learning about the magnitude of the road injuries issue, accident sites and epidemiological characteristics of the injured (e.g. young, motorcycles) - Learning about an innovative data collection tool - Envisaged improvements regarding care of the injured by the hospital and accidents monitoring by the police - Reflecting on professional practices - Individual awareness-raising on road safety
Instrumental	- Behavioural changes as a result of awareness raising, especially helmet wearing and compliance with speed limits and traffic lights - Awareness-raising of other actors about appropriate behaviours and attitudes on the road - Inspiring new ideas such as organising road safety awareness activities in the community - Using research briefs as a pedagogical tool
Persuasive	- Relaying main results in the professional environment - Using data, perceived as credible, to justify projects description and grant application - Confirming the importance of public awareness activities, especially those adapted to young people - Preparing a report after the workshop for one’s superiors - Interest in reorganising workshop so that other actors can benefit from it and enhance research dissemination - Discussing research results at a meeting with other actors in road safety, sharing research briefs, displaying researchers’ accident sites map
Process-related use	- Networking to know the actors working in the field - Creation of post-workshop collaborations - Motivation to pursue exchanges and collaboration with researchers and road safety activities - Mutual learning through interactions and deliberations - Interest of civil society to create a follow-up committee on recommendations’ implementation

Source: individual interviews with workshop participants (*n* = 14)

#### Conceptual use

The vast majority of respondents reported that the workshop provided them with new knowledge about road accidents (e.g. accident-prone zones, most affected groups, traffic light accidents, etc.). Some respondents, less involved in road safety, said the results gave them a better understanding of the magnitude of the situation. Nearly half of the respondents also mentioned having been personally made aware of the importance of wearing helmets and of their behaviour on the road. For some respondents, the study led them to reconsider their own professional practices:

“*If it hadn’t been for the workshop, if I hadn’t been made aware of the study, I would never have considered the question* [about the physical separation between the lanes for two-wheel transport and for other vehicles]*. I will discuss this with the engineer, the technical design office, and the project manager.*” (Road infrastructure worker)

#### Instrumental use

In terms of professional practices, the CSO representatives interviewed reported that they had used the information obtained at the workshop to organise and hold road safety prevention activities after the workshop. They said they wanted to integrate the study’s main messages into the training offered in road safety (e.g. driving school training for a driver’s license). As this respondent explained, the workshop also generated ideas for action at the local level:

“*Thanks to the group discussions, we were able to unleash our creativity and generate ideas,* [and to] *enrich the very content of our association’s objective by offering new awareness-raising activities in the neighbourhood.*” (Driving school instructor)

#### Persuasive use

The actors directly involved in road safety, and thus already aware of the issues, used most of the knowledge strategically. First, the results of the study confirmed the relevance of the public awareness activities on road safety carried out by the CSOs. For example, in light of the results of the study, some CSO representatives said they were motivated to intensify their efforts to promote road safety, specifically among youth. To explain the relevance of acting on road safety, some also distributed the research briefs or the main results of the study within their professional environment. Others went even further, reporting that they used the results to implement new projects by citing the results in their funding applications:

“*This year, I started writing our action plan by taking into account the data transmitted... With these data, we can update the information to justify our road safety projects.*” (CSO representative)

Several respondents reported concrete changes in their behaviour since the workshop. For example, a few respondents stated they were raising awareness about road safety in their networks.

#### Process-related use

Some pointed out that involving stakeholders during the study would have encouraged direct conversations among participants during the workshop and strengthened the general acceptability of the results:

“*Even though the study noted certain dysfunctions, I didn’t have the impression that there was anyone who felt threatened and who then took a position against the study.*” (Health NGO representative)

The experience of collaboration also seemed to have been positive, with a respondent explaining the intention to possibly include the information system set up by the researchers within the National Police and to continue the collaborations:

“*It showed us that accident reporting isn’t enough, we should perhaps even set up a monitoring body within the Police... and try to see how we might bring Police representatives and researchers together annually.*” (Police representative)

### Follow-up and implementation of recommendations

Several respondents indicated that national coordination of road safety activities would be insufficient to ensure follow-up of the various activities and ensure coordination among the different stakeholders. ONASER, the representative of the Burkinabè state, was recognised by the actors as the structure that should assume this role. However, some said ONASER did not adequately perform its mandate as intermediary between the various ministries involved in road safety and the actors on the ground. Moreover, the difficulty experienced by associations in trying to obtain funding, via ONASER, to implement road safety interventions was often noted as a major obstacle to implementing the recommendations. However, respondents were optimistic that the recent creation of a Directorate-General for Road Safety within the Ministry of Security would generate a surge of road safety actions:

“*The main obstacle is the financing of activities. It must be said that road safety is a matter of State policy. The government must make this a priority. With the recent reorganisation of ministries, this will no longer be ambiguous.*” (Representative of a government structure)

### Creating a monitoring committee

Respondents agreed, however, on the relevance of creating a monitoring committee at their level that would, for example, convey the workshop’s conclusions to the right people. While the initiative to create this monitoring committee did not materialise in the short term following the workshop, the proposal was considered useful. To prevent the scattering of efforts, it is essential to avoid any proliferation of road safety groups. The challenge is to bring together actors who are motivated to contribute to this committee and have the resources to influence change. With each person caught up in their own professional commitments, it is difficult to find people who would be dedicated to monitoring. Respondents commented on the mission that this committee could have and on its leadership. In terms of the committee’s composition, it was considered important that all stakeholders be represented to maximise its impact. However, opinions differed on the committee’s leadership. Some felt this committee should be coordinated by the researchers, since they started the initiative by convening the workshop. On the other hand, many believed the leadership should come from the CSOs, given that a structure was already in place (FAPSER), insofar as their capacity would be strengthened and resources provided to carry out the recommendations. Another proposal was to rely on neutral actors, such as knowledge brokers, who are not already implementing road safety projects. The committee would then be seen as a platform of partners:

“*The ideal would be to create a group, so that the people who were present or the institutions represented at the workshop could meet regularly to continue reflecting and see how, in their own service, they would want, or be able, to implement actions.*” (NGO representative)

Generally speaking, the mission of this committee should be to monitor and evaluate the implementation of recommendations within the various structures. Some respondents also proposed that this committee should have an advocacy mission vis-à-vis the authorities and ONASER. This committee could also take on a facilitation role, bringing together the various structures and partners by organising discussion meetings, for example. Finally, several respondents proposed that this committee could serve as a centre of expertise by providing the most recent research knowledge to stakeholders such as by offering training to CSOs.

## Discussion

Road accidents are a public health issue requiring an intersectoral approach [18]. The deliberative dialogue workshop model used as a KT strategy in Ouagadougou responded to this need. Participants recognised the usefulness of this workshop format to take advantage of their own experiential knowledge, from numerous actors, while using the knowledge produced by the study. The main effects of the workshop were at the individual level (knowledge acquisition, development of collaborations, improved understanding of the issue, etc.), with organisational changes requiring more time and perseverance [17]. However, participants observed some effects at the organisational level as a result of sharing their experiences, research briefs and workshop conclusions within their respective organisations (e.g. ideas for activities to be undertaken, development of action plans in their association, etc.). The results also showed that, while participants saw the usefulness of the knowledge and recommendations and intended to apply them in their professional practice, the fact that most of them had limited decision-making power (perception of low control) reduced the effects observed a few weeks after the workshop [38]. During the deliberations, the majority of participants first wanted to submit a report to the various ministries involved listing the recommendations developed. This proposal is understandable in the political and policy context of a country like Burkina Faso, where decision-making systems are often centralised and where international organisations and donors influence the agenda-setting for certain public health issues [39, 40]. This undoubtedly discourages local actors from taking initiative and exercising leadership, which are often determining factors in KT approaches [41]. More collective efforts will be needed to observe longer-term effects at the system level (road accident issues on the government agenda, appropriate funding mechanism for prevention activities, etc.) [13].

Despite the limited effects reported and the obstacles to implementing the monitoring committee, this deliberative workshop experience in Burkina Faso showed that stakeholders appreciated this process more than the traditional research dissemination workshop that had been held a few years earlier [25]. In fact, the efforts made to adapt the researchers’ language (i.e., to avoid scientific jargon) and limit their speaking time, to produce clear, concise and action-oriented research briefs [27], and to base the workshop on multiple interactions and the collective development of recommendations, were strengths of the approach. Thus, the majority of participants interviewed said that they would like to see a repeat of the consultation framework offered by the workshop, as this would encourage the exchange of ideas and lead to improved practices. The climate of trust fostered by the collaborative research project [42] was conducive to interaction and discussion. Participatory or embedded research is often cited as a promising approach for producing locally relevant evidence, thereby fostering better KT to improve health systems [43].

The evaluation results uncovered what may have negatively influenced the outcome of this KT approach. With reference to some of the key characteristics identified in Boyko et al.’s [17] conceptual model (Fig. 1) to foster an appropriate environment for deliberative workshops, the following reflections are aimed at better understanding how the workshop’s effects could be improved in the Burkina Faso context.

### Timeliness of the policy issue

According to Yehia and El-Jardali [44], a deliberative process is appropriate when the issue is important and in the public interest, when there is research or knowledge available on how to resolve it, and when there is openness to change in the system. In the case of road safety in Burkina Faso, the timing of the workshop was determined by the conclusion of the research project grant. This timing coincided with the end of a transitional government in the country and the holding of presidential elections a few days later. Thus, the question arose as to whether road accidents represented a sufficiently recognised and important public health issue to warrant the hope that it would be placed on the political agenda in the short term following the workshop [41, 45]. By improving visibility and monitoring, such a deliberative workshop could, in any case, contribute to the politicisation of a societal issue.

### Pre-event work

In addition to distributing research briefs beforehand to workshop participants, it is sometimes proposed that a steering committee be created before the workshop is held [7, 46]. This committee would be representative of the various stakeholders whose involvement is key to resolving the issue; its role would include performing a stakeholder mapping analysis to identify participants to be invited to the deliberations [47]. In the case of Burkina Faso, the research team organised the workshop due to time constraints. However, since the research project was conducted in close collaboration with stakeholders, the researchers were familiar with their concerns and needs. To continue with the participatory approach of the research project, it would have been useful to invite certain key stakeholders to a pre-workshop consultation meeting to validate the research briefs, clarify and verify the acceptability of the workshop objectives, and use their networks of contacts to maximise the presence of important stakeholders [7].

### Participant commitment and rules of engagement

To further engage participants, the specific workshop objectives and desired outcomes could have been communicated more clearly to participants in the invitation so they would have a clear understanding of their role in the deliberations and the utility of the process [48]. In order to move more towards best practices for deliberative processes, the same amount of speaking time should have been offered to the different stakeholders to share their respective knowledge [7]. Moreover, as mentioned above, the sometimes-limited decision-making power of the actors present may have been an impediment to the impact of the workshop [44]. When preparation time is limited because of field constraints, it would be advisable to advance more gradually and not seek to obtain participants’ commitment to implementing recommendations as early as the first workshop [10, 17]. However, if stakeholders were better prepared before the workshop, developing an action plan would be achievable. With regard to engagement, the likely influence of the per diem culture on policy-makers’ participation in research dissemination workshops should also be noted [49]. Often associated with development aid projects in Africa, per diems are used to remunerate project stakeholders to motivate them to attend workshops and thereby promote implementation of the project [50]. However, the impact of this practice on political actors’ engagement, especially in KT activities, requires further exploration.

### Adequate resources and post-event work

To organise, implement, monitor and evaluate a deliberative process, especially for deliberative dialogues at the national level, significant resources must be mobilised [51]. In Burkina Faso, there were several gaps in terms of the resources available for monitoring. This experience underscores the importance of planning to anticipate key challenges such as ensuring leadership, funding monitoring activities and supporting participants over the long term. For example, it remains to be seen whether the fact that the workshop was a research project initiative may have influenced the follow-up, given the end of the research grant and the limited availability of the researchers in the field [13]. It is also important to be aware that stakeholder involvement can impose considerable human and financial resource burdens on the various CSOs involved. Similar difficulties in coordinating follow-up and implementation of recommendations were encountered at a national deliberative workshop organised in the field of health in Niger [52], where the obstacles observed included corruption, lack of per diems paid to the monitoring committee and absence of political will.

This KT experience in the field of road safety in Burkina Faso provides lessons to guide future initiatives of this kind. Box 2 presents the main recommendations to be considered when organising, conducting and following up on a deliberative workshop.

### Limitations of the study

This evaluation presents certain limitations. The self-report questionnaires were not validated in the Burkina Faso context. Moreover, as these questionnaires were anonymous, it was not possible to match the quantitative responses with the interview data. This would have allowed the data to be triangulated for a better understanding of the quantitative item scores. The timing of the interviews may also have influenced the validity of data. Interviews should be conducted at least a few weeks or months after the workshop to give participants time to take action. In this case, the interviews may have been conducted too early, which may have limited the observation of changes. On the other hand, the passage of time can, conversely, affect participants’ ability to recall more specific elements such as the content or conduct of the workshop. As such, it would have been useful to conduct several waves of interviews, but this was not possible due to time constraints. Social desirability bias may also have positively influenced the evaluation results, even though respondents were told their feedback was being sought to improve future KT initiatives. Finally, generalisation of the study is limited, as the effects could be different depending on the nature of the issue under study, the research results presented, the different actors involved in the process and the funding available.

## Conclusion

This study showed that it could be useful to organise more deliberative workshops to foster the translation and application of research-based knowledge in Burkina Faso. It would also be important to understand the potential effects of such workshops when they are part of a broader KT approach. An additional element that could be interesting to study, for example, would be the impact of having an intermediary in the process, such as a knowledge broker, whose role would be to provide longer-term support. In future initiatives, it would be important to give early consideration to systemic issues that might influence KT in the context, such as politicisation of the issue, the decision-making process, and the scarcity of financial, material and human resources for public health. While this study proposes recommendations, further studies are needed to clarify best practices for organising, conducting and following up on a deliberative workshop, specifically in West Africa.

## Box 1 Workshop agenda 

Presentation of the study

**Table:** 

9:00–9:15	Welcome and introduction
9:15–9:30	The importance of knowledge translation and application
9:30–9:55	Genesis of the road traffic injuries research project
9:55–10:20	Accident-prone locations in Ouagadougou
10:20–10:45	What legislation is needed for road safety in Burkina Faso?
10:45–11:10	Mortality and injuries among road users in Ouagadougou
11:10–11:35	Social autopsies to better understand the context of accidents and treatment of injuries
11:35–12:00	The research project and the way forward?

Deliberative workshop

**Table:** 

13:45–15:00	Work in four subgroups
15:00–16:00	Discussions and collective synthesis
16:00–17:00	Recommendations and action plan

## Box 2 Recommendations for conducting deliberative workshops in Burkina Faso

Organisation

- ➔ Set up a steering committee that is representative of the different stakeholders to guide and validate the organisation of the workshop
- ➔ Organise pre-workshop consultations with different groups of actors to stimulate reflection and familiarise them with the workshop format
- ➔ Ensure resources are available for monitoring and/or obtain the support of governmental/international structures for the process

Conduct

- ➔ Hold the workshop over 2 days to increase the discussions, foster uptake of the information transmitted, reflect more on the implementation of recommendations, and plan the follow-up
- ➔ Limit the researchers’ presentations to give some defined speaking time to the different actors and avoid having the leadership of the process devolve to the researchers
- ➔ Project the recommendations formulated by the participants onto a screen to encourage discussion and systematise the deliberations

Follow-up

- ➔ Make conclusions available soon after the workshop to maintain active communication among the participants, and provide support to those who would like a better grasp of the knowledge
- ➔ Encourage leadership by impartial and available intermediaries to ensure a strong presence in the field
- ➔ Use mass media to disseminate the results of the study and the recommendations coming out of the workshop to give visibility to the issue

## Abbreviations

CSO: civil society organisation
KT: knowledge translation
NGO: non-governmental organisation
ONASER: National Road Safety Office
RBE: research-based evidence
